# The effects of an HIV project on HIV and non-HIV services at local government clinics in urban Kampala

**DOI:** 10.1186/1472-698X-11-S1-S9

**Published:** 2011-03-09

**Authors:** Toru Matsubayashi, Yukari C Manabe, Allan Etonu, Nambusi Kyegombe, Alex Muganzi, Alex Coutinho, David H Peters

**Affiliations:** 1 Johns Hopkins Bloomberg School of Public Health, Baltimore, Maryland 21205, USA; 2 Institute of Infectious Diseases, Kampala, Uganda; 3 Johns Hopkins School of Medicine, Baltimore, Maryland 21205, USA; 4 London School of Hygiene and Tropical Medicine, London, UK

## Abstract

**Background:**

HIV/AIDS is a major public health concern in Uganda. There is widespread consensus that weak health systems hamper the effective provision of HIV/AIDS services. In recent years, the ways in which HIV/AIDS-focused programs interact with the delivery of other health services is often discussed, but the evidence as to whether HIV/AIDS programs strengthen or distort overall health services is limited. The aim of this study was to examine the effect of a PEPFAR-funded HIV/AIDS program on six government-run general clinics in Kampala.

**Methods:**

Longitudinal information on the delivery of health services was collected at each clinic. Monthly changes in the volume of HIV and non-HIV services were analyzed by using multilevel models to examine the effect of an HIV/AIDS program on health service delivery. We also conducted a cross-sectional survey utilizing patient exit interviews to compare perceptions of the experiences of patients receiving HIV care and those receiving non-HIV care.

**Results:**

All HIV service indicators showed a positive change after the HIV program began. In particular, the number of HIV lab tests (10.58, 95% Confidence Interval (C.I.): 5.92, 15.23) and the number of pregnant women diagnosed with HIV tests (0.52, 95%C.I.: 0.15, 0.90) increased significantly after the introduction of the project. For non-HIV/AIDS health services, TB lab tests (1.19, 95%C.I.: 0.25, 2.14) and diagnoses (0.34, 95%C.I.: 0.05, 0.64) increased significantly. Noticeable increases in trends were identified in pediatric care, including immunization (52.43, 95%C.I.: 32.42, 74.43), malaria lab tests (1.21, 95%C.I.: 0.67, 1.75), malaria diagnoses (7.10, 95%C.I.: 0.73, 13.46), and skin disease diagnoses (4.92, 95%C.I.: 2.19, 7.65). Patients’ overall impressions were positive in both the HIV and non-HIV groups, with more than 90% responding favorably about their experiences.

**Conclusions:**

This study shows that when a collaboration is established to strengthen existing health systems, in addition to providing HIV/AIDS services in a setting in which other primary health care is being delivered, there are positive effects not only on HIV/AIDS services, but also on many other essential services. There was no evidence that the HIV program had any deleterious effects on health services offered at the clinics studied.

## Background

### HIV/AIDS in Uganda

HIV/AIDS has been a major cause of morbidity and mortality in Uganda since the disease was first diagnosed in early 1980s [1]. Although Uganda is often cited as a country that successfully reduced the incidence and prevalence of HIV/AIDS after peaking in the early 1990s [2][3], a recent report suggests that the prevalence rose from 6.2% in 1999/2000 to 7.7% in 2004/2005 in rural Uganda, and the same study reports that the incidence has levelled off, or has even shown signs of increasing [3,4]. In Uganda, HIV/AIDS programs are funded primarily by Western donors. The recent global economic downturn has also affected the sustainability of these programs [5], has drawn attention to the necessity of focusing more efforts on HIV/AIDS and has resulted in more calls to increase support from donor countries and global health initiatives (GHIs), primarily the Global Fund to Fight AIDS, Tuberculosis and Malaria and the U.S. President's Emergency Plan for AIDS Relief (PEPFAR) [6].

### HIV/AIDS Programs and health systems

Although HIV/AIDS has been a critical concern in many Sub-Saharan African countries including Uganda, there is widespread consensus that weak health systems hamper the effective provision of HIV/AIDS services. To improve the health of people across low- and middle- income countries, the health system will have to be addressed holistically [7,8]. In recent years, a number of global health leaders and experts have highlighted how HIV/AIDS-focused programs interact with other health services that are not included in the more targeted HIV/AIDS programs [9,10][11]. Some argue that concentrating efforts on HIV/AIDS should be able to strengthen health systems in general [12] by ensuring that qualified health care workers are at clinics, enabling resources allocated for HIV/AIDS to spill over to other health services, and drawing attention to deficiencies in the health system [13][14]. Others argue that a negative association between HIV/AIDS programs and other services exists; that the additional requirements of the HIV/AIDS programs disrupt basic health service delivery and overburden a fragile health system [15]. However, there is little evidence examining whether HIV/AIDS programs effectively strengthen health services or divert resources from other programs and distort overall health services [14,16,17]. In particular, quantitatively interpretable studies that examine the interactions between delivery of HIV/AIDS and non-HIV services have been very few, and findings have been inconclusive [18-20].

We sought to examine the effect of a PEPFAR-funded HIV/AIDS program on six local government-run, public, urban clinics in Kampala Uganda. Herein we present an analysis of the longitudinal trends of the variety of primary health care services provided at the six urban clinics, including both HIV/AIDS and non-HIV/AIDS services. Our purpose was to assess how the HIV/AIDS project may have influenced HIV/AIDS services at the clinics, as well as other primary health care services. In addition to assessing the volume of services, the results of exit interviews were conducted to explore client perceptions of the care they received.

## Methods

### HIV/AIDS project background

Mulago-Mbarara Teaching Hospitals’ Joint AIDS Program received funding from PEPFAR in May 2006 to support six urban and peri-urban primary health clinics run by the Kampala City Council (KCC) with the aim of providing sustainable antiretroviral treatment (ART) through a partnership with the Infectious Diseases Institute (IDI) and Makerere University College of Health Sciences (MakCHS). IDI has expertise in treatment, training and systems capacity building related to HIV/AIDS. Initially, IDI was providing care for many of the patients served by the KCC clinics. In order to decongest the Mulago Hospital Complex and, specifically, the IDI clinic, the IDI sought funding to support HIV/AIDS program development and care in the KCC clinics. IDI had no direct relationship with the KCC or the clinics prior to the project. Another key goal of the partnership was to enhance the responsiveness of IDI and MakCHS to the needs of the community and key organizations in civil society. This project represented an opportunity to provide more coherent care to the community, to build capacity and to forge meaningful relationships with an important local stakeholder. Reflecting this philosophy, the HIV/AIDS projects launched at the six KCC clinics attempted to work through, and improve, existing service delivery and administrative systems rather than set up parallel systems.

### Study design and location

A longitudinal design was used for the analysis of health services. More specifically, information on monthly volumes of different health services was collected for the 12 months prior to the initiation of the HIV/AIDS programs until December, 2009 (up to 36 months of follow-up for the clinics with the longest intervention time). The primary health care services that had been provided at these clinics included maternal and child health services (*e.g*., childhood immunizations; acute treatment of malaria, pneumonia, and diarrhea; antenatal care; family planning), tuberculosis testing and treatment and other basic outpatient care.

The programs supported by the IDI included the following activities (see the study by Nankumbi et al. in this issue for more details) [21]:

• Providing training and technical assistance for clinic management through both classroom training and on-site supervision and mentorship.

• Strengthening pharmacy management and technical skills in addition to effecting physical space and organizational improvements.

• Strengthening clinic management capacities to monitor patients on ART, both clinically and through lab-based diagnostics and testing.

• Providing procurement support for drugs and supplies through training in supply chain management, provision of buffer stocks and transport facilitation.

• Providing laboratory support through physical laboratory renovation and provision of new equipment.

• Ensuring that HIV testing and counseling are always available through salary top-ups of staff and provision of adequate supplies.

• Expanding HIV treatment, including ART services and PMTCT (Prevention of Mother to Child Transmission) facilitated by Protecting Families against HIV/AIDS (PREFA).

• Expanding TB screening and treatment through staff training, provision of supplies and integration with HIV care.

Six clinics (Komamboga, Kawala, Kiswa, Kisenyi, Kiruddu, and Kitebi) received funding for these activities. The clinics cover geographically distinct parts of urban Kampala and serve different communities. The programs were introduced to the clinics at different times, ranging from January 2006 at Kiswa to June 2008 at Komamboga.

As part of the study, a cross-sectional survey utilizing patient exit interviews was also conducted in July and September 2009 in order to explore patient perceptions of their experiences at the clinics. Patients were recruited from the Kiswa, Kiruddu and Kisenyi clinics as these had been receiving support from the IDI for the longest period of time. To minimize disruption to the clinics and delay to the patients, interviews were conducted as exit interviews using pre-printed forms. As such, consenting individuals were consecutively recruited until the day's target (or the end of the clinic hours) was reached. Participants were recruited and interviewed by IDI peer educators at each clinic. The inclusion criteria for the patient survey were that an individual was at least 18 years old, was able to consent to take part, and that he or she had visited the clinic on at least three separate occasions prior to the interview. Interviews were conducted in either English or Luganda, depending on the individual’s preference. The patient sampling strategy was to ensure that a representative sample of both HIV positive (both on ART and ART naïve) and HIV negative patients from each clinic were polled. General clinic and HIV clinic interviews were conducted on different days, informed by the day(s) on which the HIV clinic was held. The number of HIV-positive patients, both on ART and ART naïve was known, and 10% of the overall population of these patients was sampled. However, the total number of patients in non-HIV care at the clinics was not known. Consequently, three non-HIV care patients were sampled for every patient who was receiving HIV care.

### Study instruments and dependent variables

The health service information was collected by utilizing forms developed and used by the Ministry of Health for the Health Management Information Systems (HMIS) to avoid duplicate reporting and additional burdens on the clinic staff [22]. The HMIS instrument was designed to collect monthly data from individual clinics. The data used for this study were compiled in December 2009. The variables chosen for our study involve the following categories of services:

• HIV health services, TB health services, Malaria health services

• Maternal and Child Health (MCH)

• Family planning

• Diagnoses of dermatological problems

We selected “the number of patients provided with contraceptive injections” as an indicator for family planning because this method of contraception is widely utilized in Uganda [23], and it requires clinics to store the pharmaceutical product and carry out an actual procedure, both of which could be sacrificed if time or resources of clinic staff were diverted because of HIV/AIDS projects. The number of dermatological disease cases was selected for both adults and children because it was very common in the community in non-HIV patients.

The patient interview questionnaire was developed separately for this project. After voluntary consent was obtained, patients who received HIV and non-HIV care were asked questions with respect to their satisfaction with the medical care and the medical providers. The instrument was designed to assess patients’ clinic usage and satisfaction with the specific services they had used. The interview was designed to last no longer than 10 minutes. Informed by the project objectives and existing patient satisfaction assessment tools [24], a three-point Likert scale was used to assess patients’ agreement with a variety of statements related to their treatment experience.

### Analysis

The primary goal of the analysis was to estimate changes in monthly health services volumes after the program by the IDI was initiated. The null hypothesis was that changes in the volume of non-focal health services would have no relationship to the changes in HIV/AIDS health service delivery. Alternatively, if health services at the primary health care clinics were strengthened in a general way as a result of the project, then we could expect there to be parallel increases in volume.

All the health service data were transferred from the study instruments to Microsoft Excel (Microsoft Corporation, Redmond, WA) and translated into STATA format. Errors in each variable were checked by describing the range, identifying missing values and outliers and using graphical descriptions such as histograms and boxplots. After this process, project staff went back to the clinics to correct any errors by examining the logbooks of clinic activities when these available. When a variable was highly skewed or heavily tailed, data transformation was used so that parametric statistical techniques could be conducted smoothly.

The key feature of the dataset was that the information recorded every month at each clinic was in a clustered and longitudinal format, violating the regression assumption of the independence of observations. To account for the non-independence of observations, as well as to incorporate the inherent heterogeneity in different clinics, we developed a series of random coefficient models by using the STATA command xtmixed [25,26]. A spline term was introduced to the time variable to indicate the beginning of the project in order to estimate the change in health service volumes before and after the start of HIV/AIDS programs [27]. We specified the following random coefficient model to estimate the trend in mean numbers of service volumes per month at a typical clinic before and after the HIV program began:

*y_ij_* = *(β_1_* + *ς_1j_)* + *(β_2_* + *ς_2j_)×time_ij_* + *β_3_×(time_ij_ – time when project began)*^+^ + *ε_ij_*

Here, *y_ij_* is the estimated value of a service volume for occasion *i* and clinic *j*; *time* indicates months from the start of the observation; *ς_1j_* is a random intercept; *ς_2j_* is a random coefficient; *ε_ij_* is the residual; *β_1_* is an estimate of the baseline volume of a health service; *β_2_* is an estimate of the change in the volume of a health service per month before the HIV program; *β_3_* is the coefficient for the spline variable, representing the change in slope from the previous period; and the sum of *β_2_* + *β_3_* is the slope for the period after the HIV program began. Additional Poisson regression models were also tested in under the assumption that the outcome data represent counts, but the results do not change the inferences presented here.

For model visualization, clinic-specific predicted lines for health services outputs were produced by using empirical Bayes predictions. Categorical variables in patient exit interviews are presented as frequencies (percentages), and the comparison was made with the use of chi-square tests.

All statistical tests were two-sided and p<0.05 was considered significant. All the analyses were conducted by using STATA11.1 (StataCorp, Texas, USA).

### Study approval

Approval for this study was obtained from the IDI Scientific Review Committee, the Makerere University Research and Ethics Committee, local IRB and the Uganda National Council for Science and Technology.

## Results

**Figure 1:**
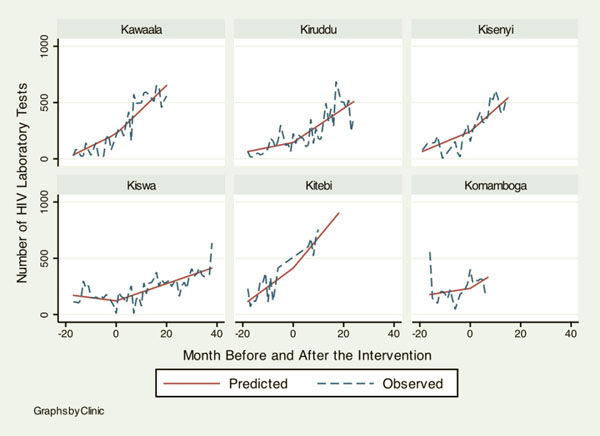
**Fitted trends for key health services.** Graphs by Clinic.

**Figure 2:**
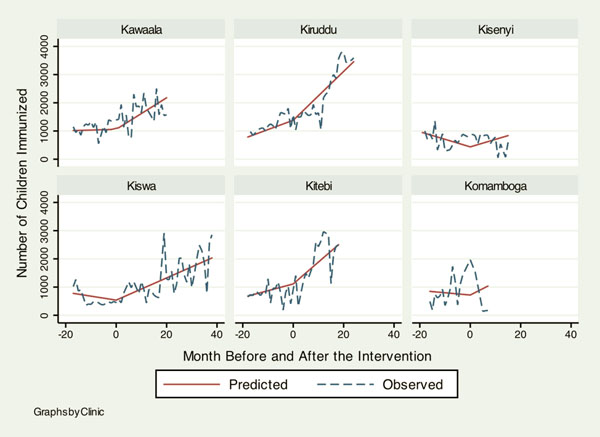
**Fitted trends for key health services.** Graphs by Clinic.

**Figure 3:**
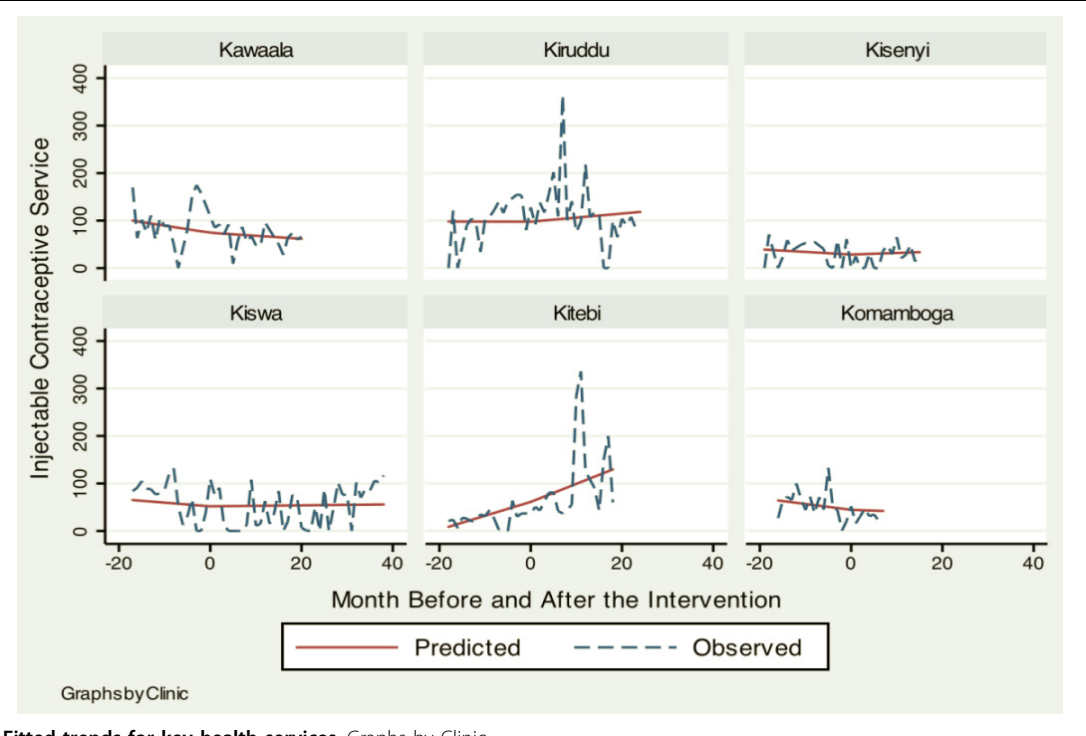
**Fitted trends for key health services.** Graphs by Clinic.

**Figure 4:**
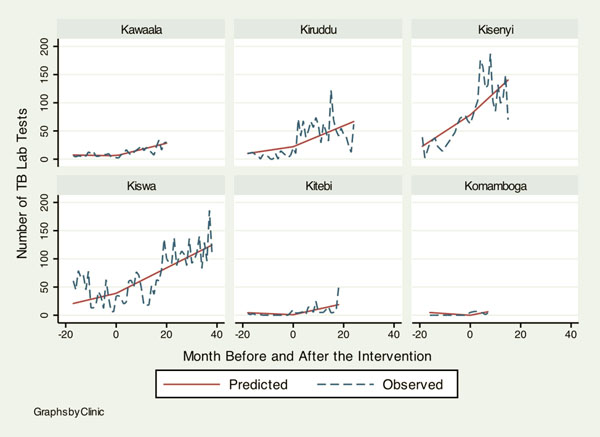
**Fitted trends for key health services.** Graphs by Clinic.

Table 1 shows baseline information on health services delivery at the six KCC clinics when the HIV/AIDS programs by the IDI were rolled out for the first time. Although overall attendance was similar across clinics, there was a noticeable heterogeneity in baseline characteristics. Notably low outputs in HIV services at Kiswa indicate that KCC clinics had very little capacity to deliver HIV services before 2006. Table 2 summarizes how monthly trends in different health services changed after the HIV programs were initiated. Figures 1, 2, 3, 4 illustrate the model-based fitted lines for key service indicators at each clinic.

**Table 1:** Baseline number of health services provided in the month before the project started

	Clinic					
	Komamboga	Kawaala	Kiswa	Kisenyi	Kiruddu	Kitebi
**Start of the HIV Project:**	**Jun-08**	**Jun-07**	**Jan-06**	**Oct-07**	**Jan-07**	**Aug-07**
**Health Services**						
**General**						
Total attendance	3162	2793	2453	2594	3761	4584
**HIV Services**						
Number of HIV Lab tests	402	271	9	153	226	415
Number of HIV positive cases	39	44	1	20	44	76
Number of HIV positive cases put on co-trimoxazole prophylaxis	6	29	1	5	25	91
PMTCT service: Number of pregnant women positive for HIV	2	0	6	3	9	30
PMTCT Service: Number of HIV positive pregnant women given ARVs for treatment	0	0	2	0	9	2
**Family Planning**						
Family Planning: Injectable Contraceptives	52	85	110	61	128	36
**Maternal and Child Health**						
Total number of children immunized	1968	1412	451	794	1579	915
Total number of pregnant women immunized	167	189	114	126	302	70
MCH: antenatal care	75	224	88	100	197	424
MCH: postnatal care	6	72	1	2	41	13
Number of pregnancy tests	0	33	0	0	0	22
**Malaria Services**						
Malaria lab tests: adults	58	120	522	196	483	54
Malaria lab tests: children	17	0	0	0	0	0
Malaria diagnosis: adults	698	803	681	1126	1450	766
Malaria diagnosis: children	447	363	334	300	875	293
**Skin Diseases**						
Number of skin disease cases: adults	93	112	98	190	409	43
Number of skin disease cases: children	56	42	57	59	155	35
**TB services**						
Number of TB lab tests	5	2	34	63	20	10
Number of TB diagnoses	0	1	5	29	10	15

**Table 2:** Monthly trend in delivery of health services at a clinic level

	Before Intervention	After Intervention	Change	95% C.I.*	p value**
Health Services					
**General**					
Total attendance	27.62	38.08	10.46	(-25.73, 46.66)	0.571
					
**HIV Services**					
Number of HIV Lab tests	7.16	17.74	10.58	(5.92, 15.23)	<0.001
Number of HIV positive cases	1.66	2.32	0.66	(-0.54, 1.87)	0.283
Number of HIV positive cases put on Septrin prophylaxis	1.58	7.09	5.51	(-0.61, 11.63)	0.078
PMTCT service: Number of pregnant women positive for HIV	0.46	0.99	0.52	(0.15, 0.90)	0.007
PMTCT Service: Number of HIV positive pregnant women given ARVs for treatment	0.36	0.48	0.12	(-0.20, 0.44)	0.476
					
**Family Planning**					
Family Planning: Injectable Contraceptives	-0.19	0.69	0.88	(-1.26, 3.02)	0.421
					
**Maternal and Child Health**					
Total number of children immunized	1.69	55.12	53.43	(32.42, 74.43)	<0.001
Total number of pregnant women immunized	-0.64	9.69	10.33	(3.30, 17.35)	0.004
MCH: antenatal care	2.72	3.63	0.91	(-0.87, 2.68)	0.316
MCH: postnatal care	0.49	0.80	0.30	(-0.86, 1.47)	0.609
Number of pregnancy tests	0.23	2.22	1.99	(0.70, 3.29)	0.003
					
**Malaria Services**					
Malaria lab tests: adults	-1.82	0.57	2.39	(-3.19, 7.97)	0.401
Malaria lab tests: children	0.03	1.23	1.21	(0.67, 1.75)	<0.001
Malaria diagnosis: adults	-1.86	8.27	10.13	(-2.87, 23.13)	0.127
Malaria diagnosis: children	0.12	7.22	7.10	(0.73, 13.46)	0.029
					
**Skin Diseases**					
Number of skin disease cases: adults	3.56	3.44	-0.13	(-3.54, 3.29)	0.941
Number of skin disease cases: children	1.00	5.92	4.92	(2.19, 7.65)	<0.001
					
**TB services**					
Number of TB lab tests	0.70	1.89	1.19	(0.25, 2.14)	0.013
Number of TB diagnoses	-0.07	0.28	0.34	(0.05, 0.64)	0.023

Monthly Changes in Health Services Per Clinic

* C.I.: Confidence Interval

** Both 95% CI and p value were for the differences of monthly changes in health services outputs between before and after the start of the HIV programs, estimated by random coefficient models.

### HIV service indicators

All variables in this category showed a positive change after intervention by the IDI began. In particular, the number of HIV lab tests increased significantly with the introduction of the project (10.58, 95%C.I.: 5.92, 15.23) and the number of pregnant women diagnosed with HIV also showed a statistically significant increase (0.52, 95%C.I.: 0.15, 0.90). One of the key PMTCT indicators, the number of women treated with anti-retroviral agents (ARVs), did not show a significant change in its trend with the project; however, the monthly output of this service increased significantly after the intervention (0.48, 95%C.I.: 0.21, 0.75).

### Non-HIV/AIDS health service indicators

Most indicators across different services showed a significant increase. First, TB lab tests (1.19, 95%C.I.: 0.25, 2.14) and diagnoses (0.34, 95%C.I.: 0.05, 0.64) increased significantly. This was probably attributable to direct intervention by the IDI, since addressing TB-HIV co-infection was one of the project goals. The second area of service that showed a noticeable increase in the trend was pediatric care, including immunization (52.43, 95%C.I.: 32.42, 74.43), lab tests for malaria (1.21, 95%C.I.: 0.67, 1.75), malaria diagnoses (7.10, 95%C.I.: 0.73, 13.46) and skin disease diagnoses (4.92, 95%C.I.: 2.19, 7.65). Health services that did not demonstrate a significant change between before and after the project intervention were family planning, antenatal care and postnatal care.

Figures 1, 2, 3, 4 illustrate how HIV and TB indicators showed positive change after the HIV programs were launched. However, it also demonstrates a conspicuous heterogeneity in the trend of health services outputs across clinics after the beginning of the project, suggesting that interactions between the HIV program and other health services can vary among different clinics. The figure includes a family planning indicator, “number of injectable contraceptives provided,” as an example of health services that did not show a change to the secular trend, the trend prior to the intervention.

### Patient exit survey and patients’ perceptions

A total of 2107 patients (774 at Kiruddu, 635 at Kisenyi, 698 at Kiswa) were interviewed, of whom 489 (23.2%) were receiving antiretroviral treatment (ART). Table 3 shows the result of three key questions asked at the exit interviews to explore the perceptions of their experiences. A comparison of the patients receiving HIV to those not receiving HIV care revealed that, in general, a greater proportion of the clients receiving HIV care were likely to respond positively to questions on a three point Likert scale (p<0.001). Overall, the proportion of all patients who reported positive perceptions of clinic care was high (more than 90% responding favorably about their experiences).

**Table 3:** Patients' perceptions of clinic experiences

		Receiving HIV Care	Not receiving HIV care	Total	
**Questions Asked**		**Percent (95% C.I.)**	**Percent (95% C.I.)**	**Percent (95% C.I.)**	**Pearson Chi Statistic**

Were you treated as an individual and compassionately?	Yes	95.62 (94.18, 97.06)	86.95 (85.04, 88.87)	90.38 (89.07, 91.68)	41.42 (p<0.001)
	Sometimes	2.84 (1.67, 4.00)	9.76 (8.07, 11.45)	7.03 (5.90, 8.16)	
	No	1.55 (0.68, 2.42)	3.28 (2.27, 4.30)	2.60 (1.89, 3.30)	

Were you able to ask questions?	Yes	94.47 (92.85, 96.08)	81.60 (79.39, 83.80)	86.68 (85.18, 88.18)	67.90 (p<0.001)
	Sometimes	3.35 (2.08, 4.61)	12.44 (10.56, 14.31)	8.85 (7.59, 10.10)
	No	2.19 (1.16, 3.22)	5.97 (4.62, 7.31)	4.47 (3.56, 5.39)

Do you trust skills and abilities of medical providers?	Yes	97.15 (95.95, 98.36)	91.50 (89.86, 93.13)	93.75 (92.64, 94.85)	25.94 (p<0.001)
	Somewhat	2.85 (1.64, 4.05)	7.52 (5.97, 9.07)	5.66 (4.61, 6.71)	
	No	0.00	0.98 (0.41, 1.57)	0.59 (0.24, 0.94)	

## Discussion

Using robust longitudinal techniques, our research demonstrated that the KCC-IDI HIV/AIDS projects were able to increase the volume of services provided for HIV, as well as for many non-HIV health services that the project did not specifically target. A significant positive change in volume was identified in TB services, immunization services and the malaria and dermatological services for children. These projects also did not appear to have any deleterious effect on any health services delivered at the primary health care clinics in Kampala. In addition, HIV patients showed that they were very satisfied with their experiences at the clinics, and non-HIV patients were positive about the quality of care they received as well.

The way HIV/AIDS-focused programs interact with delivery of other health programs has been discussed intensely in recent years. Some researchers voiced concerns about a negative impact of HIV programs on human resources, suggesting that excessive focus on rolling out ART could worsen health workforce constraints, and thereby undermine health systems [28,29]. In its evaluation of PEPFAR programs, the Institute of Medicine stated that physical infrastructure, clinics, laboratories, supply chain and information systems can be stretched thin by the implementation of a national HIV/AIDS program without careful planning [30]. Studies by McCoy and Druce suggest that expansion of access to ART could come at the expense of other vital health care services, in particular, maternal and child health care services [13,31].

However, most of these studies that have examined the relationships between HIV and non-HIV services have not been formally corroborated by statistically interpretable analyses. Two studies that examined the association between HIV and non-HIV services quantitatively should be highlighted: Price and colleagues’ study in Rwanda [19]; and Walton and colleagues’ work in Haiti [18]. The Rwanda study compared the aggregate quantities of health services over six months at 30 primary health centers before and after the introduction of basic HIV care and revealed no deleterious change in the volume of primary health services. The study showed a statistical increase not only in the volume of pediatric care (including childhood vaccinations), but also in reproductive health indicators such as antenatal care visits. There are two reasons why our study did not show the same result with respect to antenatal care. First, the Rwandan government implemented a strong initiative to strengthen service delivery in family planning, which probably caused the service volume increase in family planning and reproductive health concurrently with the study. An equivalent initiative did not occur in Uganda. Second, we performed a longitudinal analysis to examine “changes” in the monthly trend of health services, thereby isolating the effect of secular change that would have occurred regardless of the introduction of HIV programs. The methods used in the Rwanda study did not allow for such separation of a secular change. This difference in the analytical methods made our statistical estimates more conservative, but also more robust. The Haitian study had many similarities to our study, in that the HIV program was deliberately planned in a way that allowed integration of HIV services into primary health care. For example, their HIV program included training and capacity building not only for HIV care, but also for TB, STDs and women’s health. Utilization of the primary health clinic, pre-natal care, and childhood vaccinations showed a large increase, although a statistical interpretation was not provided by this study [18].

Exit surveys revealed that patients receiving both HIV care and non-HIV care were largely positive about their experiences. However, those receiving HIV care were significantly more satisfied. Several reasons could explain these findings. First, patients receiving HIV care might have experienced more intensive interaction with medical providers. Second, they might have felt a dramatically positive effect on their health status as a result of the start of HIV treatment. Another possibility is that patients receiving HIV care were more likely to be repeat users of the clinic who decided to return precisely because they were satisfied with the care and the staff, while those receiving non-HIV care were more likely to be coming for urgent care. In that case, a selection bias would be introduced to the cross-sectional sampling we utilized, leading directly to the observed difference between the two groups. Our study design could not address these questions. Further research utilizing mixed methods such as qualitative research and/or a cohort design would be necessary to explore this topic.

This study provides stronger evidence that an HIV-specific program, when deliberately planned to improve broader health areas, can help to strengthen primary health services more broadly. “Horizontal” elements of our HIV project, including capacity building for the clinic staff, better supply chain management, strengthening of lab functions and infrastructure and improvement in patient satisfaction are the potential mechanisms for the positive trend in non-HIV services. In addition, improvement of the clinic infrastructure attracted programs from other organizations such as Baylor University and PREFA, which most likely contributed to improving pediatric services. Nevertheless, our study also highlighted the fact that automatic positive spillover effect could not be expected, particularly in family planning and maternity health where no difference from the secular trend was observed after intervention.

The study also demonstrated that IDI was able to move effectively beyond its usual clinics and research sites to enter into a collaboration with KCC, helping to provide more coherent health services to its population while building the systems of its partner. This approach is consistent with the recent plans of the Makerere College of Health Sciences to play a more active role in Ugandan society.

Several limitations to the interpretations of our study need to be pointed out. First, it is not possible to determine a direct causality between the HIV program and the increase in the volume of other health services, since this is a retrospective study based on routinely collated health center data. Conducting a randomized, controlled study could better answer such a causality question; however, withholding an HIV service at a facility or community level is neither possible nor ethical, and therefore such an experimental study does not seem feasible. The longitudinal analysis employed in our study allows the strongest statistical inference for an observational study. Second, the findings need to be considered in context in order to evaluate their generalizability. Our project was carried out in an urban area, which had better access to supply and technical support than rural areas and many other countries in Sub-Saharan Africa. The urbanization of many sub-Saharan African countries make this urban data interesting nonetheless. IDI has since rolled out similar HIV programs in remote districts of Uganda with a view to applying the success of its experiences in the capital city. Future evaluation will answer the question of how repeatable this approach is. Nevertheless, we propose that all carefully planned HIV programs include a rigorous evaluation process not only for HIV-specific indicators, but also for non-HIV service indicators and contextual factors to conduct a valid program evaluation [32]. Third, our study focused on the indicators at the level of “outputs” instead of “outcome” and “impact” [33]; therefore, we cannot tell whether improvement in health service outputs catalyzed by an HIV program will lead to better outcomes and/or impacts. In fact, one ecological study that compared PEPFAR focus and non-focus countries in the WHO Africa Region [20] identified no difference in overall health impact in a six-year (2000 to 2006) period. Does this suggest that positive synergies at the level of health services outputs are not converted into actual improvement in health outcomes? Our research does not address this question. Characteristics of HIV programs have been changing from excessively vertical approaches to broader programs that increasingly recognize the importance of strengthening health systems [34,35]. The outcome and impact of such new approaches to HIV programs remain to be examined, and this is an area in which further research is warranted.

## Conclusions

This study shows that when a collaboration is established to strengthen existing systems to provide HIV/AIDS services in a setting where other primary health care is also provided, there are positive effects not only on HIV/AIDS services, but also on many other essential services, including TB services, pediatric care for skin diseases, immunization and malaria services. Although the secular trend for the indicators in family planning and reproductive health did not change, there was no evidence that the HIV program deleteriously affected any health services offered at the KCC clinics.

## List of abbreviations used

AIDS: Acquired Immune Deficiency Syndrome; ART: Anti-Retroviral Treatment; ARV: Anti-Retroviral Agents; CI: Confidence Interval; GHI: Global Health Initiative; HIV: Human Immunodeficiency Virus; HMIS: Health Management Information System; IDI: Infectious Disease Institute (Uganda); IRB: Institutional Review Board; KCC: Kampala City Council; MakCHS: Makerere University College of Health Sciences; MCH: Maternal Child Health; PEPFAR: (United States) President's Emergency Plan for AIDS Relief; PMTCT: Prevention of Mother-to-Child Transmission; PREFA: Protecting Families against HIV/AIDS; STD: Sexually Transmitted Disease.

## Competing interests

The authors declare that they have no competing interests.

## Authors' contributions

TM led the study design, data collection, data analysis and drafting of the manuscript. YCM led the study development, supervised the study execution and reviewed the manuscript. AE participated in study development and data collection. NK participated in developing the study instrument and designing the data collection process. AM participated in developing the project. AC supervised the HIV project at KCC clinics and helped develop the study concept. DHP led the study concept and design, supervised the study process and reviewed the manuscript. All authors read and approved the final manuscript.
